# The Experience of a Tertiary Reference Center in Central Anatolia with Children Carrying ZAP-70 Variants, Including Two Novel Variants

**DOI:** 10.1007/s10875-026-01989-0

**Published:** 2026-03-06

**Authors:** Serdar Göktaş, Ozlem Kalaycik Sengul, Şerife Erdem, Hatice Eke Güngör, Royala Babayeva, Sevgi Bilgic-Eltan, Turan Güzel, Koray Dörterler, Meino Rohlfs, Alper Özcan, Ebru Yilmaz, Musa Karakukcu, Haluk Himmet Akar, Elif Karakoc-Aydiner, Ahmet Özen, Muhsin Elmas, Türkan Patiroğlu, Muhammet E. Doğan, Hasan Baş, Naomi Taylor, Safa Baris, Christoph Klein, Ahmet Eken, Ekrem Ünal

**Affiliations:** 1 Faculty of Medicine, Division of Pediatric Immunology and Allergy, Erciyes University, Kayseri, Türkiye; 2 Mersin City Training and Research Hospital, Mersin, Türkiye; 3 Division of Pediatric Gastroenterology, Hepatology and Nutrition, Kanuni Sultan Suleyman Training and Research Hospital, Istanbul, Türkiye; 4 Division of Pediatric Gastroenterology, Hepatology and Nutrition, Istanbul Medeniyet University School of Medicine, Istanbul, Türkiye; 5 Department of Medical Biology, Faculty of Medicine, Betül-Ziya Eren Genome and Stem Cell Center (GENKOK), Erciyes University, Kayseri, 38030 Türkiye; 6 Department of Immunology, School of Medicine, Kırsehir Ahi Evran University, Kırsehir, Türkiye; 7 Leyla Medical Center and Republic Pediatric Center, Baku, Azerbaijan; 8 Faculty of Medicine, Department of Pediatrics, Division of Allergy and Immunology, Marmara University, Istanbul, Türkiye; 9 Istanbul Jeffrey Modell Diagnostic and Research Center for Primary Immunodeficiencies, Istanbul, Türkiye; 10 The Isil Berat Barlan Center for Translational Medicine, Immunodeficiency Research and Application Center, Istanbul, Türkiye; 11 European Academy of Allergy and Clinical Immunology, Marmara University Hospital Center of Excellence, Istanbul, Türkiye; 12 Division of Pediatric Hematology-Oncology & Pediatric HSCT Unit, Faculty of Medicine, Erciyes University, Kayseri, Türkiye; 13 Department of Pediatrics, Faculty of Medicine, Erciyes University, Kayseri, Türkiye; 14 Department of Pediatrics, Dr. von Hauner Children’s Hospital, University Hospital, Ludwig-Maximilians-University Munich, Munich, Germany; 15 Department of Blood Banking and Transfusion Medicine, Health Science Institution, Erciyes, University, Kayseri, Türkiye; 16 Division of Pediatric Immunology and Allergy, Kanuni Sultan Suleyman Training and Research Hospital, Istanbul, Türkiye; 17 Department of Medical Genetics, Medipol Mega University Hospital, Istanbul, Turkey; 18 Clinic of Medical Genetics, Kayseri City Hospital, Kayseri, Türkiye; 19 Department of Medical Genetics, Intergen Genetics and Rare Diseases Diagnosis Research & Application Center, Ankara, Türkiye; 20 Institut de Genetique Moleculaire de Montpellier, University of Montpellier, CNRS, Montpellier, France; 21 Pediatric Oncology Branch, Center for Cancer Research (CCR), National Cancer Institute (NCI), NIH, Bethesda, MD USA; 22 Department of Immunology and Microbiology, University of Colorado Anschutz Medical Center, Aurora, CO USA; 23 Medical Point Hospital, Pediatric Hematology and Oncology Clinic, Gaziantep, Türkiye; 24 Department of Pediatrics, German Center for Child and Adolescent Health (DZKJ), Munich Site, Munich, Germany

**Keywords:** Combined immunodeficiency, Novel variants, ZAP-70 deficiency

## Abstract

**Purpose:**

Zeta-chain-associated protein kinase 70 (ZAP-70) deficiency, a rare form of combined immunodeficiency (CID), is caused by homozygous or compound heterozygous variants in the *ZAP70* gene. ZAP-70, a tyrosine kinase, plays a key role in T-cell receptor (TCR) signaling, which is critical for T cell activation. ZAP-70 deficiency manifests clinically in a variety of ways, including recurring respiratory infections and cutaneous manifestations.

**Methods:**

This study describes the clinical, genetic, and immunological characteristics of four Turkish, two Syrian, and one Azerbaijani patient with ZAP-70 deficiency, including two novel variants.

**Results:**

Among seven patients diagnosed with ZAP-70 deficiency, two previously unreported ZAP70 variants were identified. Functional analyses performed in four patients—including three with novel variants—demonstrated impaired TCR-induced proliferation, reduced Interleukin 2 (IL-2) production, and markedly diminished CD8⁺ T cell numbers, supporting the pathogenicity of these variants. Clinical phenotypes were heterogeneous, ranging from severe early-onset infections and cytopenias to autoimmune manifestations and atopy. Notably, even siblings carrying the same variant exhibited divergent immunological profiles and disease severity, highlighting the influence of potential genetic or environmental modifiers. Hematopoietic stem cell transplantation (HSCT) was curative in four patients, while one patient died before transplant.

**Conclusion:**

This report expands the genetic and phenotypic spectrum of ZAP-70 deficiency by describing two novel variants and emphasizing the value of functional analysis in variant classification and patient management.

**Supplementary Information:**

The online version contains supplementary material available at 10.1007/s10875-026-01989-0.

## Introduction

*Zeta-chain-associated protein kinase 70 (ZAP70)* gene is located on chromosome 2q11.2^1^ [1]. The gene contains two non-coding and twelve coding exons that encode the 619-amino acid ZAP-70 protein [2]. It is composed of two Src Homology 2 (SH2) domains and a carboxy-terminal kinase domain separated by inter-domains A and B [3]. Loss-of-function variants of the *ZAP70* gene result in combined immunodeficiency (CID) and most are located in the kinase domain [4]. ZAP-70 is a member of the spleen tyrosine kinase (SYK) family and plays a crucial role in T-cell receptor signaling [5]. The T cell does not possess intrinsic enzymatic functions associated with the TCR or CD3 proteins. As a result of the accumulation of TCRs on the cell surface, CD3 proteins undergo conformational changes, which play a critical role in signal transduction. As soon as T cells are stimulated, the Src family of protein tyrosine kinases (PTK) (LCK and FYN) are activated. Substrates are the immunoreceptor tyrosine-based activation motifs (ITAMs) of CD3. The phosphorylation of ITAM tyrosines allows these residues to bind to other proteins with SH2 domains. The most critical SH2 domain-containing protein is ZAP-70. Activation of ZAP-70 triggers tyrosine phosphorylation of multiple substrates, resulting in the production of secondary messengers involved in T cell activation. Biallelic loss-of-function variants of the *ZAP70* gene cause proteins to be unstable and/or incapable of performing their normal functions. Consequently, affected individuals demonstrate a profound depletion of CD8 + T cells, while CD4^+^ T cell counts remain normal or elevated. However, residual CD4^+^ T cells are functionally impaired, exhibiting markedly reduced proliferation in response to CD3- or antigen-mediated stimulation [6]. This dysfunctional T cell compartment results in an increased susceptibility to bacterial, viral, and fungal infections. Additionally, serum immunoglobulin levels exhibit significant variability among patients. Clinically, ZAP-70 deficiency typically manifests within the first two years of life as a combined immunodeficiency characterized by recurrent infections, dermatologic involvement, diarrhea, lymphoproliferation, and autoimmune manifestations. In contrast to classical severe combined immunodeficiency (SCID), these patients often present with lymphocytosis and may exhibit a milder or more variable phenotype, which can lead to delayed diagnosis, especially for those with hypomorphic variants [1, 7]. ZAP-70 deficiency was first identified in the Mennonite population and has subsequently been reported in Hispanic, Japanese, Turkish, and Portuguese families [8, 9]. Here, we describe seven patients with genetically confirmed ZAP-70 deficiency, including three individuals with two previously unreported variants.

## Methods

### Study Design and Ethics

The study was conducted at the Division of Pediatric Hematology and Oncology and the Pediatric Hematopoietic Stem Cell Transplantation Unit of Erciyes University Children’s Hospital, a tertiary-care center in Central Anatolia, Turkey. For the normal value range of lymphocyte subgroups, Turkish studies were taken as reference in order to be compatible with the patient population [10, 11]. The Erciyes University Ethics Committee granted permission for a review of all records (number: 2025/281 date:28.05.2025).

### Genetic Analysis

Whole Exome Sequencing (WES) was performed for all patients. The procedure utilized Agilent V5 + UTR library preparation and was sequenced on an Illumina NextSeq 500 platform. The bioinformatics analysis pipeline incorporates several advanced tools and databases: Sequence Alignment: Burrows-Wheeler Aligner (BWA 0.7.15) was used for aligning the sequencing reads. Variant Calling: The Genome Analysis ToolKit (GATK 3.6) facilitates genetic variant identification. Variant Annotation: Variant Effect Predictor (VEP 89) was employed for annotating the variants. Frequency Filtering: Variants were filtered based on frequency using both public and in-house databases, including Exome Aggregation Consortium (ExAC), Genome Aggregation Database (gnomAD), and Greater Middle East Variome (GME). Selected variants are classified according to American College of Medical Genetics and Genomics (ACMG) criteria [12]. To confirm the identified genetic variants in the patients and assess segregation within the family, Sanger sequencing was performed.

### Cell Isolation

Peripheral blood mononuclear cells (PBMCs) were isolated from EDTA-anticoagulated peripheral blood samples obtained from both healthy controls and ZAP-70–deficient patients using the Ficoll-Paque Plus (GE Healthcare) density-gradient centrifugation method. The viability and density assessment of PBMCs was conducted manually using a Thoma slide and Trypan Blue stain prior to the experiment. In all experiments, cells were resuspended in Stain Buffer [phosphate-buffered saline (PBS) with 2% fetal bovine serum (FBS)], and an equivalent amount of cells was utilized for each condition. Every experimental condition was conducted with a minimum of three technical replicates.

### Flow Cytometry

To identify T lymphocyte phenotypes, PBMCs were treated with CD3 (clone UCHT1), CD4 (clone RPA-T4), and CD8 (clone RPA-T8) for surface staining at 4 °C for 30 min in darkness. Following staining, cells were rinsed and subsequently analyzed using BD FACSARIAIII flow cytometry. Data were analyzed utilizing FlowJo v10 software. The median fluorescence intensity (MFI) values for CD3, CD4, and CD8 surface molecules were assessed, and cell percentages were computed using FSC-A/CD3/CD4/CD8 gating. Interleukin 2 (IL-2) production from CD4⁺ T cells was evaluated by stimulating PBMCs with anti-CD3 (clone OKT3, 1 µg/mL) and anti-CD28 (clone CD28.2, 1 µg/mL) for 16 h. Golgi stop (eBioscience) was administered to the cells for the final four hours. Unstimulated cells served as the negative control. Following surface CD4 staining, cells were fixed and permeabilized using the Cytofix/Cytoperm kit (BD Biosciences), after which intracellular staining for IL-2 (clone MQ1-17H12) was conducted. The ratio of IL-2⁺ cells to CD4⁺ cells was assessed using flow cytometry. Peripheral blood mononuclear cells (PBMCs) were stained with CellTrace CFSE (Invitrogen, 1 µM) following the manufacturer’s protocol to assess lymphocyte proliferation. Stained cells were cultured for four days under various stimulation conditions: (i) unstimulated, (ii) anti-CD3/CD28 at 1 µg/mL each, (iii) recombinant human IL-2 at 100 IU/mL, (iv) phytohemagglutinin (PHA) at 2 µg/mL, and (v) PI [phorbol 12-myristate 13-acetate (PMA)/Ionomycin] with PMA at 50 ng/mL and Ionomycin at 500 ng/mL. At the conclusion of the fourth day, surface staining for CD3 and CD4 was performed, and the reduction in CFSE signal along with the proliferation rate was assessed using flow cytometry.

### Statistical Analysis

For Figs. 2, 3 and 4, due to low sample size and non-normal distribution, the Mann-Whitney U test was used to determine significance of differences between pairwise comparisons. A P-value < 0.05 was regarded as statistically significant. GraphPadPrism 8.0 was used for calculations. For descriptive statistics, continuous variables were reported as medians with interquartile ranges (IQR), and categorical variables were expressed as absolute numbers and percentages. No inferential statistical analyses were performed. All data tabulation and descriptive analyses were conducted using Microsoft Excel 2021 (Microsoft Office LTSC Professional Plus, Microsoft Corp., Redmond, WA, USA).

## Results

Among the seven patients, four were male and three were female. The mean age at diagnosis was 10.9 months. The cohort consisted of four Turkish, two Syrian, and one Azerbaijani patient.

### Genetic Findings

**Fig. 1:**
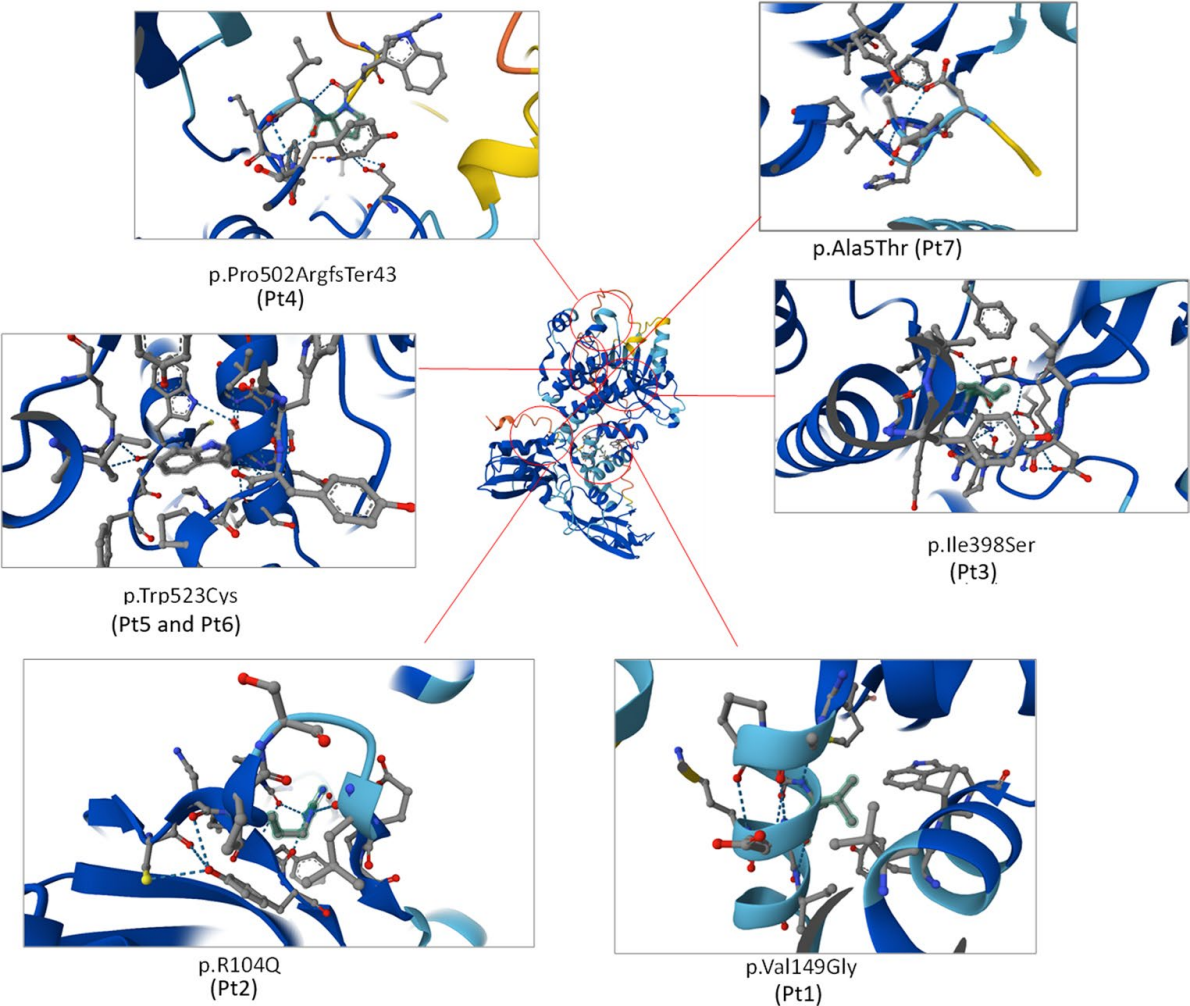
Structural representation of ZAP70 variations discovered in patients. The AlphaFold-predicted structure of the ZAP70 protein is displayed centrally, accompanied by magnified panels that emphasize the precise locations of the detected variations. Each inset depicts the atomic surroundings of the altered residue through PyMOL-generated 3D representations. Variants comprise: p.Pro502ArgfsTer43 (Pt4), p.Trp523Cys (Pt5 and Pt6), p.Ala5Thr (Pt7), p.Ile398Ser (Pt3), p.Arg104Gln (Pt2), and p.Val149Gly (Pt1). Mutated residues are represented as stick models and color-coded to differentiate modified side chain interactions. Disruption of hydrogen bonding networks and localized conformational alterations are apparent in many variations

In our cohort, six patients carried homozygous missense variants and one patient had a homozygous frameshift variant. Two missense variants—identified in Pt1 and in the siblings Pt5 and Pt6—were novel (Table 1). The positions of all variants within the protein structure are depicted in Fig. 1. Although most changes were classified as variants of uncertain significance according to ACMG criteria, their segregation patterns, absence or rarity in population databases, and supportive in silico predictions suggest potential pathogenicity.

**Table 1:** Molecular features of patients with *ZAP70* (NM_001079.4) variants

Patient	Variant	Exon	Type	Zygosity	Ethnicity	Consanguinity	Classification
Pt1	c.446T > G; p.Val149Gly	4	Missense	Hom.	Syrian	Yes	VUS
Pt2	c.1193T > G; p.Ile398Ser	10	Missense	Hom.	Turkish	Yes	Likely pathogenic
Pt3	c.311G > A; p.Arg104Gln	3	Missense	Hom.	Syrian	Yes	VUS
Pt4	c.1503_1504dup; p.Pro502ArgfsTer43	12	Frameshift	Hom.	Turkish	Yes	Pathogenic
Pt5, Pt6	c.1569G > C; p.Trp523Cys	12	Missense	Hom.	Turkish	Yes	VUS
Pt7	c.13G > A; p.Ala5Thr	3	Missense	Hom.	Azerbaijani	Yes	VUS

*Hom.* homozygous; *Pt* patient; *VUS* variant of uncertain significance

Pt1 carried a novel homozygous missense variant in exon 4 (c.446T > G; p.Val149Gly). Pt2 had a previously described likely pathogenic missense variant in exon 10 (c.1193T > G; p.Ile398Ser) [12, 13]. Pt3 carried a rare missense variant in exon 3 (c.311G > A; p.Arg104Gln), observed at extremely low frequency in population databases, and the patient’s phenotype closely aligned with previously reported cases of ZAP70 deficiency. Pt4 harbored a homozygous frameshift variant in exon 12 (c.1503_1504dup; p.Pro502ArgfsTer43), absent from population databases but previously reported in association with ZAP70 deficiency [2, 13]. Pt5 and Pt6, who were siblings, shared a novel missense variant in exon 12 (c.1569G > C; p.Trp523Cys). Pt7 carried a rare homozygous missense variant in exon 3 (c.13G > A; p.Ala5Thr).

Overall, the clinical presentation, inheritance patterns, and available functional evidence collectively support the pathogenicity of the identified ZAP70 variants.

### Immunological Findings

The most common profile among evaluated patients was low CD8^+^ cell counts (42.8%). There was neutropenia in one patient (Pt1) and lymphocytosis in another patient (Pt2). One patient (Pt1) exhibited isolated low serum IgG levels, two patients (Pt2 and Pt7) had elevated serum IgE levels, Pt6 showed reduced IgA levels, and Pt7 demonstrated concomitant reductions in both IgA and IgM. Three patients (Pt3, Pt4, and Pt7) exhibited reduced CD8⁺ T cell counts (/mm³). In addition, Pt7 demonstrated decreased CD3⁺ and CD4⁺ T cell counts, while CD16/56⁺ NK cell numbers were low in two patients (Pt2 and Pt7). All patients had normal CD19^+^ B cell counts (/mm³). Isohaemagglutinin levels were not assessed in patients under one year, as it was deemed that the results would not accurately reflect the true physiological condition. Two patients (Pt2, Pt6) exhibited low isohaemagglutinin titers (Table 2).

**Table 2:** The immunological profile of the ZAP70 deficiency patients with combined immunodeficiency

Parameters	Pt1	Pt2	Pt3	Pt4	Pt5	Pt6	Pt7
WBC×10^3^ (cells/µL), (Rv)	9,94 (5.5–17.5 × 10^3^)	16,46 (5.5-15.5 × 10^3^)	6,64 (6–17 × 10^3^)	16,34 (6–17 × 10^3^)	9,08 (6–17 × 10^3^)	14,37 (5.5–15.5 × 10^3^)	26,3 (5.5–17.5 × 10^3^)
ALC × 10^3^ (cells/µL), (Rv)	8,74 (4-10.5) x10^3^)	9,02 (2–8 × 10^3^)	3,26 (4-13.5 × 10^3^)	8,09 (4-13.5 × 10^3^)	6,53 (4-13.5 × 10^3^))	7,80 (2–8 × 10^3^)	4.99 (4-10.5) x10^3^/µl)
ANC ×10^3^ (cells/µL), (Rv)	0,87 (1–8,5 × 10^3^)	6,46 (1.5–8.5 × 10^3^)	2,9 (1.5–8.5 × 10^3^)	5,51 (1.5–8.5 × 10^3^)	1,87 (1.5–8.5 × 10^3^)	5,67 (1.5–8.5 × 10^3^)	19.82 (1–8,5 × 10^3^/µl))
IgG mg/dL, (Min–max)	205 (242–977)	2060 (389–1260)	792 (270–1110)	1500 (217–981)	239 (217–981)	712 (389–1260)	1230 (Received IVIG) (242–977)
IgM mg/dL, (Min–max)	125 (24.2–162)	234 (38.6–195)	254 (26.9–130)	257 (15.2–68.5)	21 (15.2–68.5)	55 (38.6–195)	18 (24.2–162)
IgA mg/dL, (Min–max)	60,1 (6.68–114)	235 (13.1–103)	42 (6.67–53)	311 (6.67–24.6)	7 (6.67–24.6)	13 (13.1–103)	5 (6.68–114)
IgE (U/ml)	29,3	1800	60	17,3	13,7	25	1424
CD3^+^ T cells (cells/µL), median (Ti^−^Ti^+^)	4293 (1981–6564)	3000 (1338–6611)	2532 (1492–6385)	6400 (1492–6385)	2265 (1492–6385)	5493 (1338–6611)	1209 (1945–7129)
CD4^+^ T cells (cells/µL), median (Ti^−^Ti^+^)	3560 (1190–4481)	1400 (820–4138)	2386 (909–4523)	6196 (909–4523)	1649 (909–4523)	3038 (820–4138)	1145 (1161–4819)
CD8^+^ T cells (cells/µL), median (Ti^−^Ti^+^)	674 (576–2582)	1540 (540–2812)	82 (254–2123)	194 (254–2123)	581 (254–2123)	2025 (540–2812)	55 (310–2250)
CD16/56^+^ B cells (cells/µL), median (Ti^−^Ti^+^)	1963 (156–968)	91 (101–1741)	457 (101–1633)	648 (101–1633)	1218 (101–1633)	340 (101–1741)	102 (130–1073)
CD19^+^ B cells (cell/µL), median (Ti^−^Ti^+^)	929 (117–2845)	3794 (516–3083)	1504 (237–2564)	1012 (237–2564)	1686 (237–2564)	1527 (516–3083)	3381 (467–3112)
Anti-HBS	+	-	+	-	+	+	N/A
*Isohemagglutinins*	-	-	+	+	-	-	N/A

Reference intervals for immunoglobulin and lymphocyte subsets were obtained from Turkey studies, reflecting population-specific values [10, 11]

*ALC* Absolute lymphocyte count; *ANC* Absolute neutrophil count; *Ig* Immunoglobulin; *WBC* White blood cell; *NK cell* Natural killer cell

**Fig. 2:**
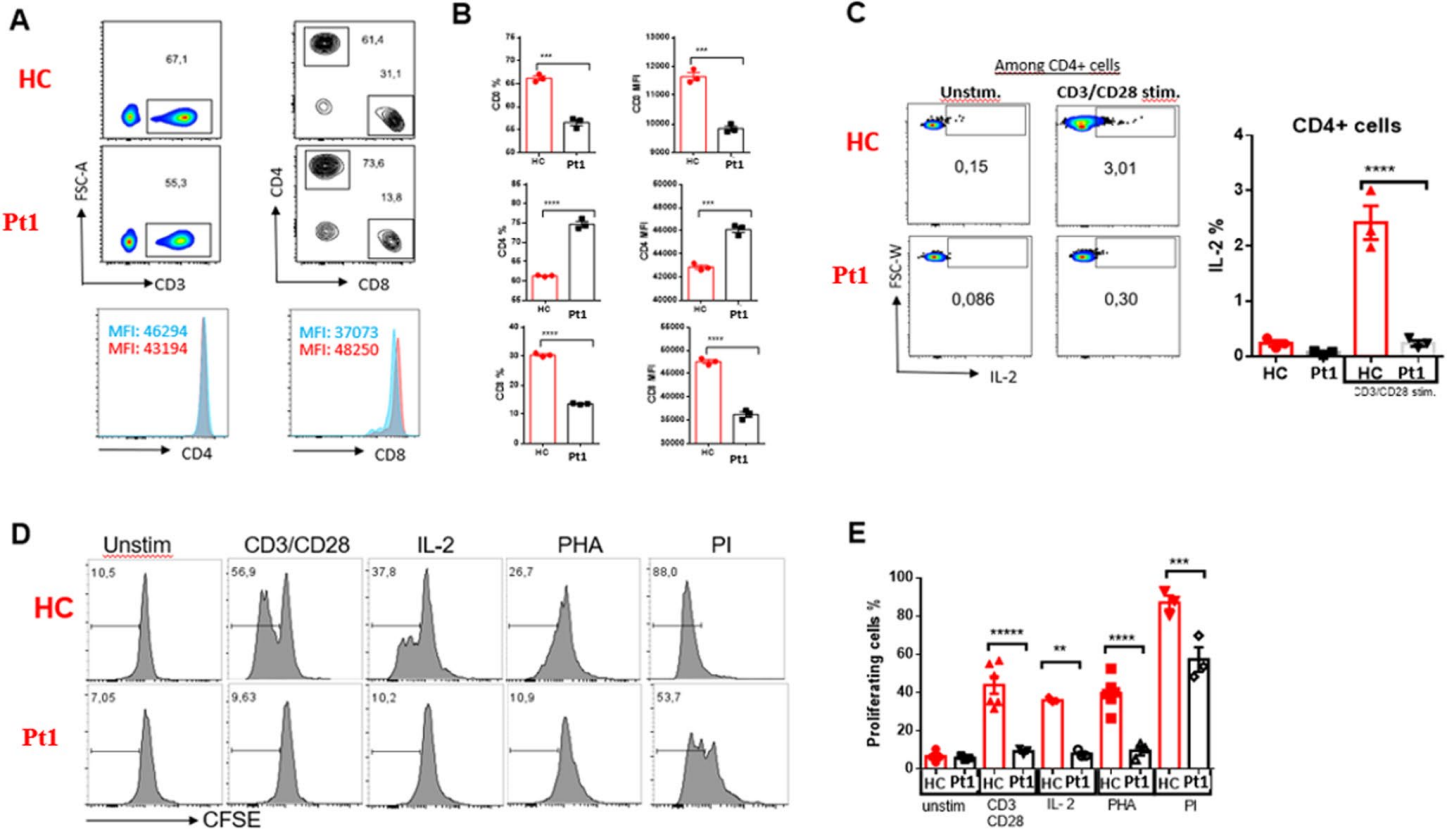
Decreased CD8⁺ T cell frequency and impaired T cell activation and proliferation in Pt1 carrying the ZAP70 variant. (**A**-**B**) Lymphocytes from both the patient (technical replicate) and controls(*n* = 3) were subjected to surface staining with anti-CD3, CD4, and CD8 antibodies. The results are presented in both (**A**) flow cytometry plots and (**B**) bar graphs. (**C**) CD4⁺ T cells were stimulated for 24 h with anti-CD3/CD28 antibodies and assessed for IL-2 production via intracellular staining. Representative flow cytometry plots (left) and summary bar graphs (right) show a marked decrease in IL-2 production by CD4⁺ T cells in Pt1. (**D**) CFSE-labeled PBMCs from Pt1 and healthy controls (*n* = 3) were stimulated with various mitogens (CD3/CD28, IL-2, PHA, PI) for 96 h. Histograms represent CFSE dilution as a measure of cell division. (**E**) Bar graphs quantify the percentage of proliferating cells (CFSE) under each stimulation condition. Pt1 exhibited significantly impaired proliferation in response to CD3/CD28, IL-2, and PHA stimulation compared to HC. Proliferation in response to PI was preserved. Data for HC are shown as mean ± SEM. Patient sample was analyzed in technical replicates. *p < 0.05; **p < 0.01; ***p < 0.001; ****p < 0.0001

We performed functional analysis on four patients (Pt1, Pt5, Pt6, Pt7), three of whom had novel variants (Pt1, Pt5, Pt6). Figure 2A shows CD3, CD4, CD8 Flow cytometry trapping graphs. Pt1 peripheral blood has statistically significant CD3 and CD8 low CD4 high percentage and MFI (Fig. 2B).

Figure 2C shows IL-2 production following lymphocyte activation. After stimulation with CD3/CD28, the proportion of CD4⁺ T cells producing IL-2 was significantly reduced in Pt1 compared to healthy control (HC). Figure 2D-E evaluates the proliferative responses of CFSE-stained T cells. In HC, T cell division was high after CD3/CD28, IL-2, PHA and PI stimulation, whereas the proliferation response in Pt1 cells was significantly lower in all conditions (Fig. 2E).

**Fig. 3:**
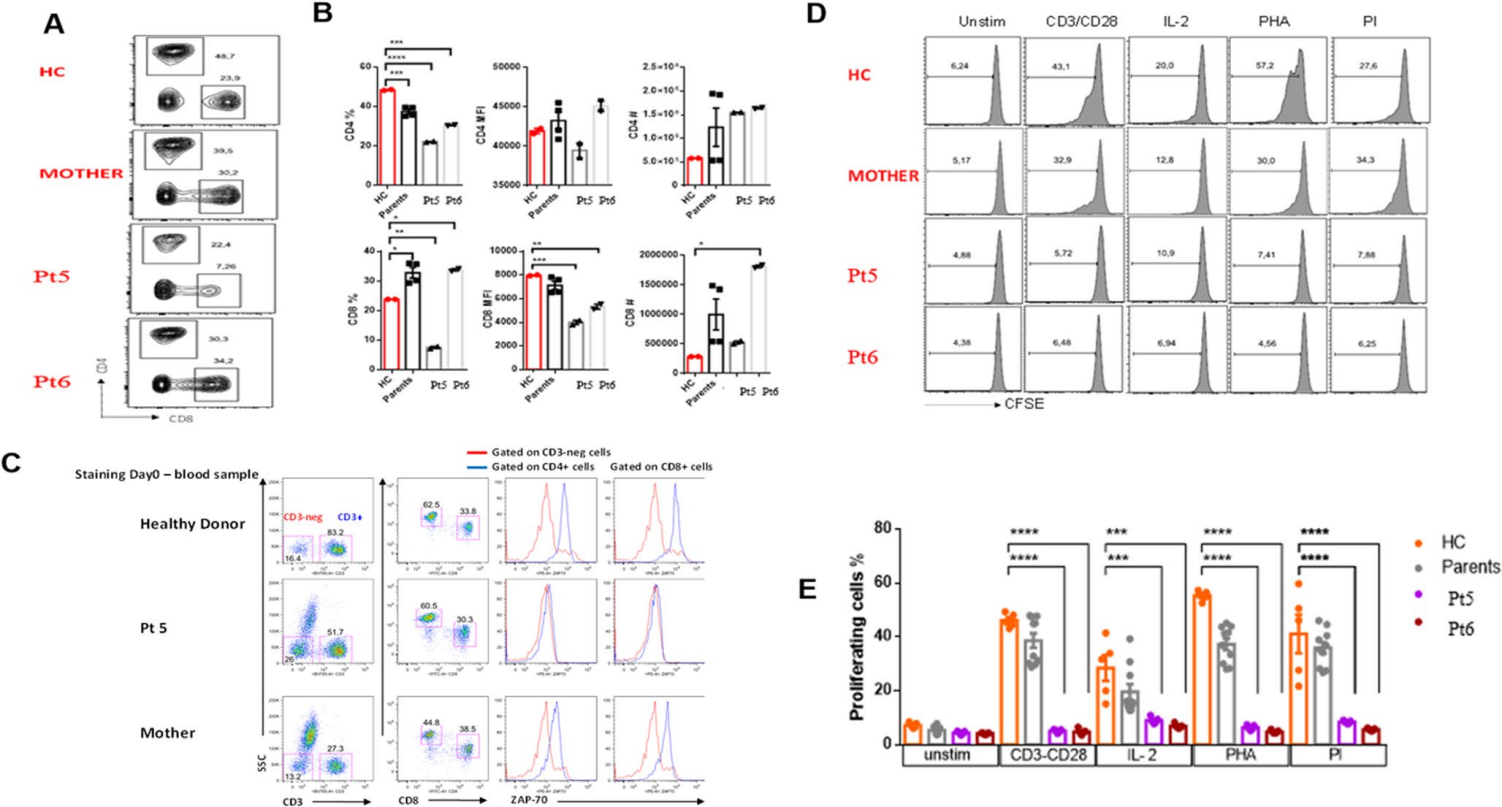
Reduction in CD8 Cell Frequency and Defect in Mitogen-Dependent Proliferation in Patients Pt5 and Pt6 with the ZAP70 variant. (**A**) Flow cytometry plots showing CD4⁺ and CD8⁺ T cell distributions in PBMCs from healthy control (HC), the mother, Pt5, and Pt6. (**B**) Quantitative analysis of T cell frequencies and CD4/CD8 expression (MFI) in all individuals. (**C**) ZAP-70 protein expression was analyzed by flow cytometry in CD4⁺ and CD8⁺ T cells. Both Pt5 and their mother exhibited decreased ZAP-70 levels. (**D**) CFSE-labeled PBMCs were stimulated with CD3/CD28, IL-2, PHA, or PI. Histograms represent CFSE dilution in T cells. (**E**) Proliferating cell percentages are plotted, showing reduced proliferation in both Pt5 and Pt6 compared to HC and the mother. HC data shown as mean ± SEM; patients and mother were analyzed in technical replicates. *p < 0.05; **p < 0.01; ***p < 0.001; ****p < 0.0001

Pt5 and Pt6 are siblings, both of which exhibit altered T cell subsets. Figure 3A shows CD4/CD8 flow cytometry captures. Pt5 showed a marked decrease in both CD4^+^ and CD8^+^ T cell percentage and MFI compared to HC, while Pt6 showed a more balanced profile with a slight decrease in CD4 + T cell percentage and an increase in CD8 + T cell percentage and MFI (Fig. 3A-B). Furthermore, one of the patients (Pt5) showed greatly reduced ZAP70 protein expression as seen by intracellular staining of CD4 and CD8^+^ T cell subsets, confirming that the variant leads to loss of protein expression/function (Fig. 3C).

Pt5 and Pt6 also exhibited markedly impaired T cell proliferation in response to various stimuli (Fig. 3D-E). In both Pt5 and Pt6, there was a significant reduction in proliferation following CD3/CD28, IL-2, PHA and PI stimulation compared to HC. Notably, the proliferation response in Pt5 was almost non-existent in all conditions, suggesting a severe defect in TCR signaling and cellular activation. Pt6 also exhibited reduced proliferative capacity, although slightly better than Pt6, especially under PI stimulation.

**Fig. 4:**
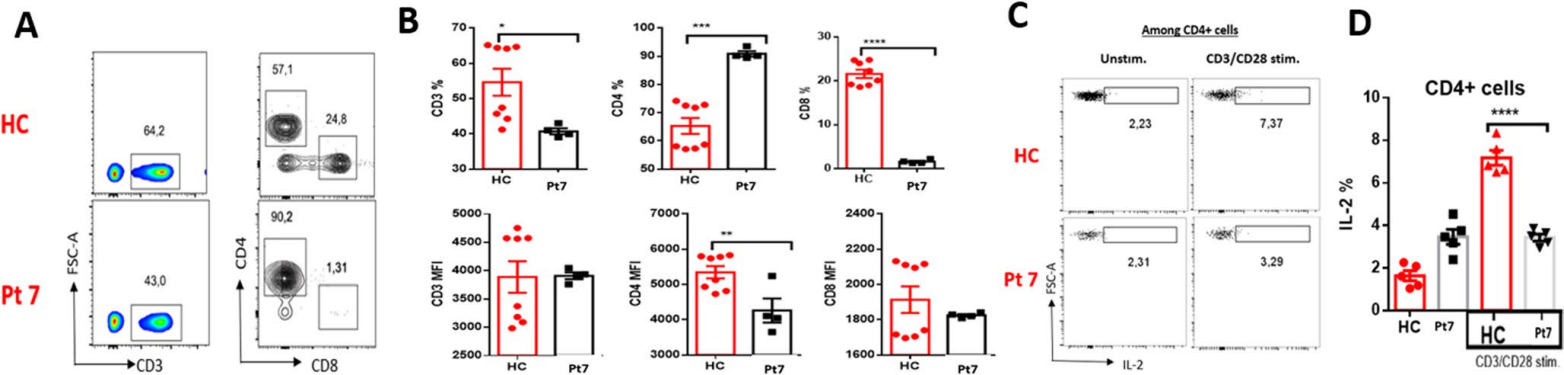
Profound reduction in CD8⁺ T cells and impaired IL-2 production in Pt7. (**A**) Representative flow cytometry plots showing CD3⁺, CD4⁺, and CD8⁺ T cell gating in HC and Pt7. (**B**) Quantification of CD3⁺, CD4⁺, and CD8⁺ T cell percentages and corresponding MFI values. Pt7 exhibited markedly decreased CD3⁺ and CD8⁺ T cell frequencies, with expanded CD4⁺ T cells and reduced CD4 expression levels. (**C**) CD3/CD28-stimulated CD4⁺ T cells were stained for intracellular IL-2. Flow plots demonstrate lower IL-2 production in Pt7. (**D**) Bar graph summarizing IL-2⁺ CD4⁺ T cells in HC and Pt7. Data represent mean ± SEM for HC; Pt7 analyzed in technical replicates. *p < 0.05; **p < 0.01; ***p < 0.001; ****p < 0.0001

A decrease in the percentage of CD3⁺ and CD8⁺ T cells and an increase in the percentage of CD4⁺ T cells was observed in Pt7 (Fig. 4A-B). Furthermore, upon CD3/CD28 stimulation, the percentage of IL-2 producing CD4⁺ T cells in Pt7 was significantly lower than that observed in healthy controls (Fig. 4C-D).

### Clinical Findings

**Fig. 5:**
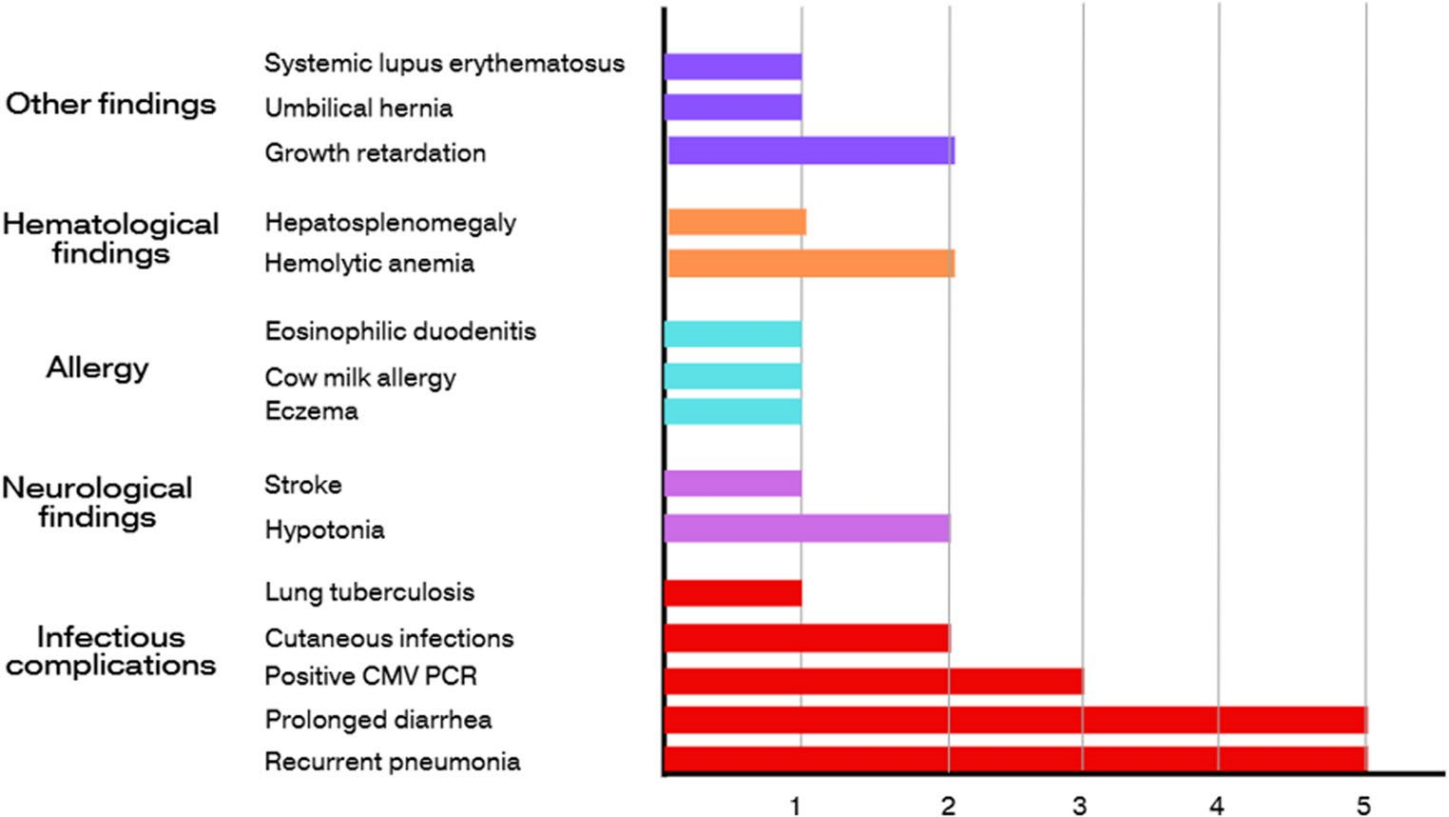
Summary of clinical features observed in patients carrying ZAP70 variants. Bar graph representing the frequency of clinical manifestations among patients with ZAP70 variants. Categories include infectious complications (e.g., recurrent pneumonia, prolonged diarrhea), neurological findings (e.g., hypotonia, stroke), allergy, hematologic findings (e.g., hemolytic anemia), and other syndromic features (e.g., umbilical hernia, SLE). Clinical features were compiled across the cohort and grouped by system

The clinical findings were diverse, involving multiple organ systems, with infectious complications, neurological abnormalities, atopic manifestations, and hematological issues being the most prominent features. All patients presented with clinical findings consistent with CID. Recurrent pneumonia and prolonged diarrhea were the most common initial presentations, each observed in 71.4% of the cohort (Table 3; Fig. 5).

**Table 3:** The clinical and follow-up characteristics of the patients

Patient ID	Age/sex	Consanguinity	Main clinical presentation	CD8(Count)*	Outcome
Pt1	7 months/M	+	Prolonged diarrhea, severe pneumonia, eosinophilic duodenitis and bulbitis, CMV infection, malnutrition, hypotony.	674(576-2582)	HSCT
Pt2	2 years/M	+	Prolonged diarrhea, frequent URTI, recurrent pneumonia, jaundice, CM allergy, hepatosplenomegaly, SLE, autoimmune hemolytic anemia	1540(540-2812)	HSCT
Pt3	5 months/F	+	Prolonged diarrhea, atopic dermatitis, cutaneous infections	82 (254-2123)	HSCT
Pt4	3 months/M	+	Tuberculosis, recurrent pneumonia, umbilical hernia, GR, stroke (MTHFR homozygote)	194(254-2123)	HSCT
Pt5	3 months/F	+	Severe pneumonia, CMV infection	581(254-2123)	Trimetoprim/sulfametoksazol, valganciclovir, IVIG
Pt6	2 years/F	+	Prolonged diarrhea, severe pneumonia, CMV infection	2025(540-2812)	Trimetoprim/sulfametoksazol, valganciclovir, IVIG
Pt7	10 months/M	+	Prolonged diarrhea, autoimmune hemolytic anemia, hypotony, GR, cutaneous infections	55(310-2250)	Exitus

*CM allergy* cow’s milk allergy; *CMV* cytomegalovirus; *F* female; *GR* growth retardation; *HSCT* hematopoietic stem cell transplantation; *IVIG* intravenous immunoglobulin; *M* male; *MTHFR* methylenetetrahydrofolate reductase; *SLE* systemic lupus erythematosus; *URTI* upper respiratory tract infection

#### Infectious Complications

Both recurrent pneumonia (Pt1, Pt2, Pt4, Pt5, Pt6) and prolonged diarrhea (Pt1, Pt2, Pt3, Pt6, Pt7) were each reported in five patients (71.4%). Microbiological evaluation revealed Cytomegalovirus (CMV) PCR positivity in Pt1, Pt5, and Pt6, while EBV PCR results were negative in all patients. In addition, a carbapenem-resistant Klebsiella pneumoniae strain was isolated from the rectal swab of Pt3. As a result of the Bacillus Calmette-Guerin vaccination administered at the hospital, one patient developed pulmonary tuberculosis (Pt4). Aside from these findings, no additional pathogen-specific isolates were identified in our center.

#### Neurological Findings

Two patients developed hypotonia (Pt1, Pt7), while another patient with a homozygous variant in the MTHFR gene suffered a stroke (Pt4).

#### Atopy

One patient had eosinophilic duodenitis and bulbitis (Pt1), one had a cow’s milk allergy (Pt2), and another patient had eczema (Pt3). All of these patients exhibited peripheral blood eosinophilia.

#### Hematological Findings

Autoimmune hemolytic anemia was observed in two patients (Pt2 and Pt7), while hepatosplenomegaly was present only in Pt2.

#### Other Findings

Growth retardation was observed in two patients (Pt4 and Pt7), whereas an umbilical hernia was present in one patient (Pt4). Additionally, Pt2 was diagnosed with systemic lupus erythematosus.

**Hematopoietic Stem Cell Transplantation (HSCT) Characteristics**.

Among the patients who underwent HSCT in our center, Pt1 and Pt3 were successfully transplanted according to their conditioning and prophylaxis protocols, achieving full donor chimerism in both cases. Pt1 received prophylaxis with acyclovir, micafungin, and trimethoprim–sulfamethoxazole, followed by conditioning with busulfan, fludarabine, and ATG, and GVHD prophylaxis with cyclosporine A (CSA) and methotrexate (MTX), using a fully matched (10/10) HLA-identical paternal donor. Pt3 received similar prophylaxis; conditioning included busulfan, fludarabine, ATG, and rituximab, with GVHD prophylaxis provided using CSA, and transplantation was performed using a paternal haploidentical donor (5/10 match) within an α/β T-cell–depleted platform. Although Pt3 developed post-transplant Langerhans cell histiocytosis, the disease responded well to chemotherapy and is currently in complete remission without complications.

Pt5 and Pt6 were evaluated as HSCT candidates based on immunophenotypic and clinical findings and received standard antiviral, antifungal, and P. jirovecii prophylaxis during follow-up, but neither progressed to transplantation. Pt7 had HSCT planned but died before conditioning could be initiated.

For Pt2 and Pt4, whose transplant procedures were performed in external centers, detailed information on conditioning regimens, donor characteristics, GVHD prophylaxis, and antimicrobial prophylaxis was not available. However, the accessible clinical documentation indicates that both maintained full donor chimerism and exhibited no significant transplant-related complications during long-term follow-up.

## Discussion

In this study, we aimed to describe the clinical, immunological, and molecular features of patients with ZAP-70 deficiency, including those carrying novel variants, and to evaluate the pathogenic potential of these variants through functional studies. In our cohort, CD8⁺ T cell counts were reduced in three patients (Pt3, Pt4, Pt7), consistent with the variable immunophenotypic presentation reported in ZAP-70 deficiency [14].

During infancy, patients commonly present with recurrent severe microbial infections that are indistinguishable from SCID [15–18]. Recurrent pneumonia and prolonged diarrhea were observed in the majority of patients, while CMV infection was diagnosed in 42.8% of the cohort (Pt1, Pt5, Pt6). A heterogeneous spectrum of clinical symptoms in ZAP-70 deficiency often leads to diagnostic delays [1]. In our cohort, the predominance of early-onset presentation, with a mean age at diagnosis of 10.9 months and most patients diagnosed within the first year of life, reflects the need for heightened clinical suspicion during infancy.

ZAP-70 is essential for T-cell receptor signaling, particularly for the development and function of CD8⁺ T cells and the activation of CD4⁺ T cells [4]. Combined hypomorphic and activating variants in ZAP-70 have been associated with severe autoimmunity, normal or reduced CD4⁺ T and B cells, normal IgA levels, low IgM levels, and normal-to-low IgG levels. In our cohort, humoral abnormalities varied across patients, including isolated low IgG levels (Pt1), elevated IgE levels (Pt2, Pt7), reduced IgA levels (Pt6), and combined reductions in both IgA and IgM (Pt7). Because CD4⁺ T cells in ZAP-70 deficiency are functionally impaired, they cannot provide adequate help to B cells for immunoglobulin production, leading to defective antibody responses and, in many cases, hypogammaglobulinemia. Consistent with this, systematic reviews and case reports indicate that a substantial proportion of individuals with ZAP-70 deficiency demonstrate defective antibody production (57%) and hypogammaglobulinemia, often accompanied by poor vaccine responses and recurrent infections [4, 19].

Among the seven patients with ZAP-70 deficiency in our cohort, two novel *ZAP70* variants (Pt1, Pt5, Pt6) were identified. Due to the small number of ZAP-70 deficiency patients and striking heterogeneity in their clinical presentation, it was difficult to identify any association between the type and location of variant and disease course or outcome in patients who showed similar reductions in ZAP-70 expression [20].

For Pt1, Pt3, Pt5, Pt6, and Pt7 the course of disease reported herein provides compelling evidence that the variants identified in them, although classified as VUS, are most probably disease-causing. More detailed interpretation is possible for the four patients (Pt1, Pt5, Pt6, Pt7) who underwent functional studies. In ZAP-70 deficiency, immunological evaluations typically show normal T cell counts and CD4 + T cell counts, but a significant decrease in CD8^+^ T cell counts. Antigen-specific proliferation is impaired. IL-2 production and proliferation in response to PHA and anti-CD3/CD28 antibodies are impaired [21].

Functional evaluations performed on four patients (Pt1, Pt5, Pt6, and Pt7) revealed a consistent pattern of impaired T cell proliferation in response to CD3/CD28, IL-2, PHA, and PMA/Ionomycin stimulation, reflecting a shared TCR signaling defect. All four patients showed abnormalities in T cell subset distribution, most notably reduced CD8⁺ T cell counts. In Pt5 and Pt7, these immunophenotypic alterations were accompanied by markedly decreased surface expression of both CD4 and CD8 co-receptors, as indicated by reduced mean fluorescence intensity (MFI). Pt1 exhibited increased CD4⁺ T cell frequency along with decreased CD8⁺ T cell counts and reduced IL-2 production upon CD3/CD28 activation, despite relatively preserved MFI. Pt6 demonstrated a more balanced immunophenotype, with elevated CD8⁺ T cell percentage and expression, but still exhibited impaired proliferation. Intracellular ZAP-70 protein staining in Pt5 confirmed loss of protein expression, further substantiating the functional consequences of the variant. Collectively, these findings support the pathogenicity of the identified variants and align with previous studies highlighting impaired T cell activation and differentiation as hallmarks of ZAP-70 deficiency.

Among these patients, Pt5 and Pt6 were siblings carrying the same missense variant. While both exhibited reduced proliferative responses, Pt5 demonstrated a more severe immunological and clinical phenotype, characterized by markedly decreased CD4⁺ and CD8⁺ T-cell percentages, significantly lower co-receptor expression, and an almost absent proliferative response across all stimulation conditions, accompanied by prolonged hospitalization and the need for intensive care support. In contrast, Pt6 displayed a relatively milder immunophenotype, with preserved CD8⁺ T-cell expression and a more robust response to PI stimulation. These findings suggest that, even within the same genetic context, disease severity may be modulated by additional genetic or environmental modifiers, as highlighted in prior studies [19, 22–24].

The remaining patients, Pt2, Pt3, and Pt4, who did not undergo functional assays, also exhibited clinical phenotypes supportive of ZAP-70 deficiency. Pt2 exhibited a classic combined immunodeficiency phenotype characterized by prolonged diarrhea, recurrent upper and lower respiratory tract infections, pneumonia, and multi-system autoimmunity, including systemic lupus erythematosus and autoimmune hemolytic anemia—features highly consistent with impaired T-cell function [13, 19, 25, 26]. Pt3 displayed a predominantly cutaneous–gastrointestinal phenotype, with prolonged diarrhea, atopic dermatitis, and recurrent skin infections, reflecting disrupted T-cell–mediated immune regulation at epithelial barriers. Pt4 demonstrated the most complex clinical course, marked by recurrent pneumonia, pulmonary tuberculosis, significant growth retardation, and a history of stroke associated with homozygous MTHFR mutation; together, these manifestations underscore the profound T-cell dysfunction and broad phenotypic variability characteristic of ZAP-70 deficiency.

We could not detect any correlation between clinical and laboratory findings and the reported variants in our patients. Although genotype-phenotype correlation was not evident in our cohort, this finding is consistent with prior observations suggesting that the clinical severity of ZAP-70 deficiency is influenced not only by the variant site but also by other modifier genetic and environmental factors [19, 22–24].

Newborns with low T-cell receptor excision circles (TRECs) or CD8^+^ T cell count, consanguineous parents, and a positive family history of ZAP-70 deficiency should be screened for this condition. Monitoring sibling Pt6 for a known ZAP70 gene defect led to Pt5’s diagnosis.

Valganciclovir was administered to Pt1 before HSCT due to CMV PCR positivity, and it is also currently being used for Pt5 and Pt6 for the same indication. Both patients are also receiving intravenous immunoglobulin (IVIG) as supportive therapy. For patients with ZAP-70 deficiency, allogeneic HSCT remains the only curative treatment. In our cohort, all four transplanted patients were alive and clinically well at a median follow-up of 36 months. Although early HSCT has been associated with improved outcomes in previous reports, the small sample size and limited follow-up duration in our study restrict definitive comparison with the literature [19, 27]. Pt5 and Pt6 did not have a fully matched related donor, prompting the initiation of an unrelated donor search. Whether the transplanted patients represented clinically less severe cases remains uncertain. Younger age is associated with fewer complications and better HSCT outcomes; therefore, early screening and timely HSCT may help reduce disease burden.

This study has several limitations. First, the small sample size restricts the generalizability of the findings. Microbiological data were incomplete for some individuals because infectious episodes were managed in external medical centers, preventing full access to pathogen isolation results. In addition, T-cell receptor repertoire assessment and naïve/memory T-cell phenotyping could not be performed due to the limited availability of these advanced assays across participating centers. Finally, comprehensive information on HSCT protocols and detailed long-term post-transplant outcomes could not be obtained for patients who underwent transplantation in external centers, representing a further constraint in evaluating treatment-related and follow-up data.

Despite these limitations, the extreme rarity of ZAP-70 deficiency, together with the identification of novel variants supported by functional validation, makes this cohort a valuable contribution to the expanding molecular and immunological understanding of the disorder.

In conclusion, ZAP-70 deficiency presents a diagnostic challenge because lymphocyte counts may appear normal on routine testing. Detailed immunophenotyping and genetic analysis are therefore essential for diagnosis. While allogeneic HSCT remains the only curative option, further experimental studies—including gene-based approaches—are needed to explore alternative therapeutic strategies.

## Electronic Supplementary Material

Below is the link to the electronic supplementary material.

Supplementary Material 1

## Data Availability

No datasets were generated or analysed during the current study.
