# Supplementary material for: The Experience of a Tertiary Reference Center in Central Anatolia with Children Carrying ZAP-70 Variants, Including Two Novel Variants
Source: J Clin Immunol. 2026 Mar 6;46(1):32. doi: 10.1007/s10875-026-01989-0 (PMC13004735; doi:10.1007/s10875-026-01989-0)
Supplement: Supplementary file 1 — Supplementary Material 1 [file 10875_2026_1989_MOESM1_ESM.docx]

**Supplementary Table 1. Detailed Molecular Characteristics of Identified ZAP70 Variants**

| **Patient** | **Variant** | **Exon** | **gnomad** | **Novelty** | **REVEL** | **AlphaMissense** | **PrimateAI** |
| --- | --- | --- | --- | --- | --- | --- | --- |
| Pt1 | c.446T>G; p.Val149Gly | 4 | N/A | + | 0.83  Delet. | 0.908  Delet. | 0.47  Benign |
| Pt2 | c.1193T>G; p.Ile398Ser | 10 | N/A | - | 0.88 Delet. | 0.998  Delet. | 0.76 Uncertain |
| Pt3 | c.311G>A; p.Arg104Gln | 3 | 0.0002% | - | 0.23 Benign | 0.903  Delet. | 0.60 Uncertain |
| Pt4 | c.1503_1504dup; p.Pro502ArgfsTer43 | 12 | N/A | - | N/A | N/A | N/A |
| Pt5, Pt6 | c.1569G>C; p.Trp523Cys | 12 | N/A | + | 0.81 Delet. | 1  Delet. | 0.89  Delet. |
| Pt7 | c.13G>A; p.Ala5Thr | 3 | 0.0002% | - | 0.25 Benign | 0.854  Delet. | 0.68 Uncertain |

N/A, not available; +, novel variant; –, previously reported.
